# Unsolved Puzzles Surrounding HCV Immunity: Heterologous Immunity Adds Another Dimension

**DOI:** 10.3390/ijms18081626

**Published:** 2017-07-27

**Authors:** Babita Agrawal, Shakti Singh, Nancy Gupta, Wen Li, Satish Vedi, Rakesh Kumar

**Affiliations:** 1 Department of Surgery, Faculty of Medicine and Dentistry, University of Alberta, Edmonton, AB T6G 2R3, Canada; Shakti1@ualberta.ca (S.S.); wl6@ualberta.ca (W.L.); 2 Department of Laboratory Medicine & Pathology, Faculty of Medicine and Dentistry, University of Alberta, Edmonton, AB T6G 2R3, Canada; gupta1@ualberta.ca (N.G.); vedi@ualberta.ca (S.V.); rkumar@ualberta.ca (R.K.)

**Keywords:** hepatitis C virus (HCV), adenoviruses, heterologous immunity

## Abstract

Chronic infection with hepatitis C virus (HCV) afflicts 3% of the world’s population and can lead to serious and late-stage liver diseases. Developing a vaccine for HCV is challenging because the correlates of protection are uncertain and traditional vaccine approaches do not work. Studies of natural immunity to HCV in humans have resulted in many enigmas. Human beings are not immunologically naïve because they are continually exposed to various environmental microbes and antigens, creating large populations of memory T cells. Heterologous immunity occurs when this pool of memory T cells cross-react against a new pathogen in an individual. Such heterologous immunity could influence the outcome when an individual is infected by a pathogen. We have recently made an unexpected finding that adenoviruses, a common environmental pathogen and an experimental vaccine vector, can induce robust cross-reactive immune responses against multiple antigens of HCV. Our unique finding of previously uncharacterized heterologous immunity against HCV opens new avenues to understand HCV pathogenesis and develop effective vaccines.

## 1. Introduction to Hepatitis C Virus (HCV) Infection

**Figure 1:**
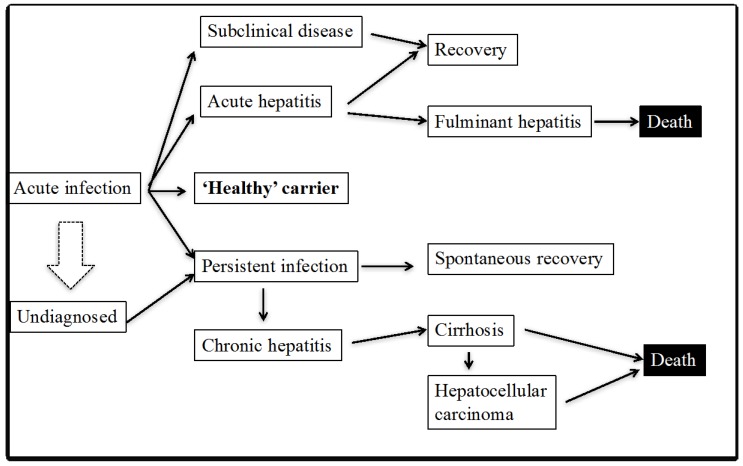
Variations in clinical course of Hepatitis C virus (HCV) infections.

Approximately 3% of the world’s population is chronically infected with hepatitis C virus (HCV) [1]. HCV is an important pathogen infecting more than 175 million people worldwide leading to chronic infections which can result in liver cirrhosis, hepatocellular carcinoma, liver damage, and end-stage liver disease (Figure 1). Infection with HCV is generally subclinical, and is rarely cleared with an effective immune response. In >70% of infected people, chronic disease follows the initial infection. However, >50% of chronic patients are unaware of their infection [2]. The majority of chronic HCV patients are seropositive (antibody positive), implying that induced immune responses are inefficient—that this virus is able to modulate immune responses and/or that they facilitate chronic pathological consequences in the majority of infected individuals [3]. Effective cellular immune responses have been suggested to play an important role in acute clearance of the virus and provide long-term protection [4]. Development of a vaccine against HCV has been challenging, as it is not clear what kind of immune response is needed and how to induce it, despite intense basic and clinical research in the last 25 years [5]. The development of direct-acting antivirals has dramatically changed the fate of chronic carriers, provided that they (a) are actually diagnosed; (b) are eligible to receive the drugs; (c) respond to, can tolerate, and can afford the treatment; (d) do not develop viral resistance; and (e) do not get re-infected [6]. With these limitations, antivirals cannot curb or end the HCV epidemic in the large population that is afflicted globally. The need for a vaccine remains [7].

## 2. Enigmas of HCV Immunity

**Figure 2:**
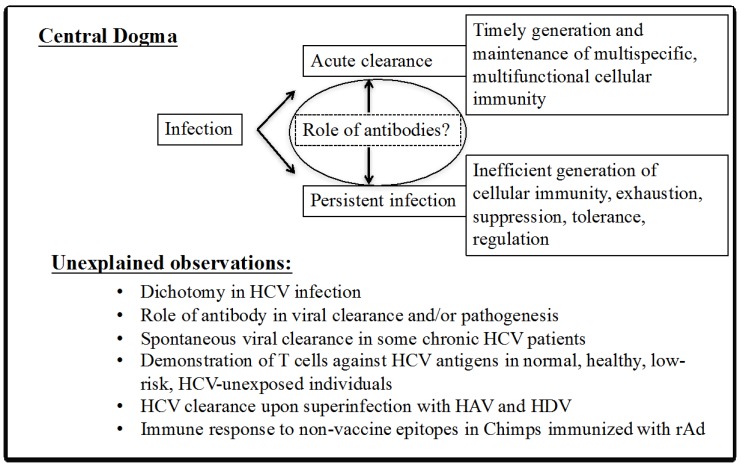
Enigmas of HCV immunity. Abbreviations: HCV, hepatitis C virus; HAV, hepatitis A virus; HDV, hepatitis delta virus; rAd, recombinant adenovirus.

It has been suggested that natural immunity dictates two courses of HCV infection: acute self-clearing or chronic persistent (Figure 2). The mechanisms behind this dichotomy are still unclear, however. It has been suggested that the acute self-clearing HCV infection is associated with timely induction of multi-specific and multi-functional T cell responses against HCV antigens [8]. There are still many unexplained paradoxes: (a) only 20–30% of the HCV-exposed individuals clear HCV infection due to rapid emergence of strong and multi-specific immune responses; (b) some chronic HCV patients spontaneously clear the virus; (c) HCV-specific T cells can be expanded from blood of previously unexposed individuals; (d) T cells in rAd (recombinant adenovirus vector)-vaccinated chimps can target HCV epitopes not present in the rAd vaccine itself [9,10,11,12,13,14,15,16,17,18]. Some specific examples are described as follows. In one retrospective study examining 10,318 chronic hepatitis C patients, 50 cases of late spontaneous viral clearance were observed, and the clearance was found to be associated with female sex, young age at infection, lower HCV RNA load, and co-infection with hepatitis B virus, however, no clear explanation of spontaneous viral clearance was provided [10]. In a prospective study with 139 patients with chronic hepatitis C infection, a significant percentage of patients demonstrated a spontaneous negative HCV RNA test result, corresponding to a clearance rate of 1.15 cases per 100 persons per year and approx. 50% of these spontaneous clearance cases were durable [11]. Immunologic determinants of spontaneous clearance were not studied in both of these reports. In a study with a chronic HCV infected patient with late spontaneous clearance (at >65 weeks), sudden appearance of both humoral and cellular response against HCV was associated with viral clearance [12]. While the trigger of this immune response was not clear, it was suspected that heterologous viral infections leading to induction of effective HCV immunity could be a mechanism. The authors ruled out hepatitis B virus (HBV) and human immunodeficiency virus type-1 (HIV-1) infections in this subject and found no significant change in Epstein Barr virus (EBV)- and cytomegalovirus (CMV)-specific T-cell responses, and in influenza A virus–specific T-cell and antibody responses, eliminating a possible cross-reactivity with these infections [12]. Therefore, spontaneous viral clearance in chronic HCV patients largely remains as an unexplained observation. In addition, there have been a number of reports demonstrating successful detection of rather high frequencies of HCV specific T cell responses from HCV-unexposed/uninfected individuals [13,14,15,16,17]. In a very recent report, among a total of 121 HCV seronegative patients, ~33% of the individuals demonstrated a high prevalence of a single epitope of HCV (NS3-1073) specific CD8^+^ T cell responses upon ex vivo stimulation [15]. A possibility of nonspecific staining by the reagents was eliminated, but the origin and reason for the existence and abundance of HCV-specific T cells with a memory phenotype in HCV-seronegative risk-free persons with no history of HCV exposure could not be explained. The authors suggested that “the presence of NS3-1073-specific CD8 T cells in seronegative individuals displaying a memory phenotype can also be explained by the cross-reactivity of memory cells specific to unrelated pathogens” (page 8314), but provided no specific explanation. In another study, the HCV epitope NS3-1073 was found to be cross-reactive with an epitope derived from influenza virus neuraminidase (19). In an earlier study published in the year 2006, using a large panel of synthetic peptides covering the whole HCV polyprotein (601 peptides), multiple peptides reactive T cells were found in the blood of HCV-negative adults without risk factors of HCV infection such as blood transfusion, sexual contact with HCV-infected subjects, and intravenous drug use. Interestingly, in HCV negative subjects, HCV peptide reactive CD4 and/or CD8 T cells were detected and the frequency of peptide reactive T cells was comparable to HCV-specific T cells present in subjects who recovered from HCV infection. In addition, HCV-peptides’ specific T cells expanded and produced Th1-like cytokines interferon-gamma (IFN-γ), tumor necrosis factor-alpha (TNF-α) but not interleukin-4 or 5 (IL-4 or IL-5). Thus, the peptide-specific T cell responses identified from HCV-unexposed individuals were indistinguishable from classical virus-specific effector/memory T cells present in patients with resolved viral infection [16]. Degenerate T cell reactivity due to some heterologous infection was suspected but not clearly identified. Another interesting observation made was that the overall quantity and the frequency of HCV-peptide reactive T cells was even higher in HCV-negative people than in chronically HCV-infected patients [16]. The authors found homology in one specific HCV-peptide with a human herpes virus type 1 (HHV-1) derived peptide, but no explanation was provided for multi-peptide cross-reactivity. This comprehensive study using overlapping synthetic peptides from HCV again indicated towards a common heterologous infection as being the cause of induction of multi-specific T cell responses against HCV, but failed to identify it. In another study published in 2007 using HCV-uninfected adults, it was shown that a number of peptides derived from HCV antigens NS3 and core were highly permissive in binding to multiple human leukocyte antigen (HLA) II molecules and stimulated CD4^+^ T cells from uninfected healthy donors [17]. The authors did not suspect cross-reactivity and predicted a high frequency of naïve T cells. Corroborating these studies, a study published in 2012 demonstrated that 20% of the HCV uninfected individuals demonstrated substantial T cell responses against HCV antigens [14]. The authors proposed two theories to support their observations: inefficacy of the current diagnostic tests to detect low level infections with HCV which is sufficient to prime T cells or existence of cross-reactive T cell epitopes from other pathogens [14]. Interestingly, in chimpanzees primed with rAd containing NS3-NS5 antigens of HCV, followed by boosting with plasmid DNA, T cell responses were generated which responded to heterologous HCV peptide sequences and protection to heterologous HCV viral challenge [18]. Although the reason was not clearly explained, it could be possible that the heterologous immune responses induced by the Ad vaccine vector added to the heterologous protection. Therefore, although limited, earlier studies have demonstrated an apparent unexplainable heterologous immunity taking part in HCV immunity.

It is not known if pre-existing heterologous immunity has any role in pathogenesis or viral clearance in HCV infection. A single cross-reactive T cell epitope identified between HCV NS3 and influenza A virus may not be sufficient to influence the course of HCV infection [19]. It has been demonstrated that super-infection of HCV infected patients with hepatitis A virus was associated with spontaneous clearance of HCV in two patients [20]. In addition, acute self-limited infection with HBV/HDV led to spontaneous clearance of HCV in a chronically infected patient [21] and chronic hepatitis delta virus infection is associated with higher frequency of HCV clearance [22]. The mechanisms leading to these events have not been clearly delineated, but are speculated to be caused by the bystander effect of inducing IFN-α [23]. This speculation is unclear, since studies in a chimpanzee model of HCV infection have not shown a correlation of HCV clearance with IFN-α induction in the liver [24]. 

## 3. Adenoviruses, Ubiquitous Pathogens and Vaccine Vectors

Adenoviruses (Ad) belong to a diverse family (>50 serotypes) of DNA viruses called adenoviridae. They are non-enveloped, icosahedral viruses containing a double-stranded DNA genome and infect ocular, respiratory, or gastro-intestinal epithelium in a diverse range of hosts [25]. Some Ad have proven to be safe and efficient vaccine vectors in many animal and human clinical studies, by eliciting protective immune responses against a transgene antigen [26,27]. It is commonly known that the neutralizing antibodies against Ad5 are prevalent in humans, and vaccine vectors based on alternative serotypes or species are in development, with the speculation that pre-existing immunity against these would be rare in humans [28]. A chimpanzee-derived adenovirus, expressing HCV antigens NS3-NS5, is currently in clinical trials [29]. However, due to conserved epitopes present in proteins of various adenoviruses, even Ad5-exposed humans have been shown to have T cells cross-reactive against divergent serotypes of Ad from both humans and chimpanzees. These include Ad26, 35, 48, AdC6, and AdC7 [30]. How cellular immunity to Ad affects vaccine-induced immune responses remains to be established, but it can be speculated that there would be both positive and negative effects. On the one hand, pre-existing memory T cells against adenoviruses can boost the responses generated against the vaccine vector, but on the other hand, cytotoxic T cells generated against a previous adenovirus exposure can result in rapid clearance of adenovirus antigen-expressing cells, leading to reduced vaccine efficacy. Aside from the impact on the vaccines’ efficacy, there has been no report on the induction of heterologous immunity against other pathogens by Ad.

## 4. Evolution of an Unexpected Finding: Heterologous Immunity between Adenoviruses and HCV

Our group has published a series of papers demonstrating efficient priming of HCV antigen-specific T cells from HCV-naïve individuals, by using autologous dendritic cells transduced with recombinant adenovirus vectors (rAds) that possessed various HCV antigens [13,31,32]. More importantly, we could demonstrate a clearly enhanced response to HCV antigens when T cells were primed with recombinant Ad containing HCV antigens, compared to non-recombinant adenovirus [13,31,32]. At the time these studies were conducted, we did not have any suspicion of cross-reactivity between HCV and Ad, nor was there evidence or even conjecture of cross-reactivity in the literature. Consequently, we have been rather puzzled by the ease with which we could prime the apparently naïve human T cells from HCV-unexposed individuals against multiple epitopes of multiple HCV antigens, as well as the apparent high frequency of naïve T cells that respond to HCV antigens and the apparent dichotomy of the quality of T cells induced in vitro [31]. At the same time, our efforts to prime naïve human T cells in vitro against antigens of other pathogens have resulted in less robust and rather modest results (B. Agrawal et al., unpublished results). Studies of heterologous immunity to other pathogens using human T cells are complicated, but studies using mouse T cells provide rather clear and conclusive results [33]. For example, it has been shown in C57bl/6 mice that infections with Bacille Calmette Guerin (BCG), influenza A virus (IAV), lymphocytic choriomeningitis virus (LCMV), murine cytomegalovirus (MCMV), and Pichinde virus (PV) all confer a level of protective immunity against vaccinia virus (VV), but these have been difficult to demonstrate in humans [33]. Supporting this notion, our studies in mice led to the unexpected but conclusive demonstration of heterologous immunity between HCV and adenoviruses [34]. We were surprised to find that all published studies that examined the induction of HCV antigen-specific immunity using recombinant adenoviruses that expressed specific HCV antigens did not use non-recombinant adenoviruses as controls but rather used no-immunization (or phosphate buffered saline (PBS) immunization) as controls. This precluded them from making the unexpected finding of heterologous immunity [35]. In our initial work, we also followed this trend and used PBS-immunized mice as controls [30,31]. During these studies, immunization with recombinant Ad expressing NS3 and NS4 antigens of HCV led to the expected responses against the target antigens [36,37]. However, our groups’ efforts to generate T cells against core, F protein, and NS5A antigens of HCV using a recombinant adenovirus vector were futile (Unpublished results and [38]). Unexpectedly, however, immunization of mice with non-recombinant, replication incompetent Ad5 vector not only gave T cell responses against core and NS5A, but also robust responses against NS3 and F antigens of HCV. In contrast, NS4 antigen appeared to be less immunogenic in C57/bl6 mice [36,37,38]. Moreover, immunization of mice with recombinant Ad containing foreign non-HCV antigens such as Ag85A and Ag85B of mycobacteria, Gag antigen of HIV, and glycoprotein (GP) antigen of Ebola virus, all led to generation of T cells that responded to HCV antigens in addition to responding to the antigen of the transgene (B. Agrawal et al., unpublished results). Why did other groups not observe this cross-reactivity and what could be the reason for this robust multi-specific T cell cross-reactivity? The answer for the first question, besides mouse studies where non-recombinant Ad have not been used as controls, comes from a human Phase 1 clinical trial with chimp Ad containing NS3–NS5. Here the subjects with preexisting humoral immunity to Ad were excluded. Whether they had T cells that responded to HCV proteins was not investigated [29]. To answer the second question, we performed extensive in silico studies evaluating homologies between HCV and Ad proteins [34]. Our approach was remarkably minimalist in that we looked for homologies in peptide sequences between 15–20 aa long from various HCV antigens and 27 different Ad proteins. Our approach was significantly different than what other researchers commonly use and possibly contributed to the demonstration that there were very high homologies, ranging from 25–53% for various peptides of HCV to aa sequences of Ad proteins [34]. Comparing the entire genome or aa sequences of HCV polyprotein and Ad may not have led to the identification of specific homologies, in contrast to comparing small peptide sequences of HCV with entire aa sequences of various Ad. To our surprise, peptides derived from core, F protein, NS3, NS5 antigens of the HCV antigens demonstrated significant homology (25–53%) to one or more of the Ad proteins, whereas NS4 demonstrated lower homology (Table 1). HCV antigens E1, E2, P7, and NS2 showed low homology (<25%) with the lowest number of Ad proteins and are not shown in the table. Interestingly, we did not find any epitope of HCV with more than 53% homology to aa sequences of Ad proteins [34]. One might argue that such levels of homology may not be sufficient to induce cross-reactive T cells between Ad and HCV. To resolve this incongruity, the principles of T cell recognition of peptides in context of major histocompatibility complex (MHC) molecules have to be revisited [39,40], and to facilitate this concept, Table 2 demonstrates direct comparisons of selected HCV core peptides with the identified homologous peptide sequence of Ad5 protein. 

## 5. Revisiting T Cell Receptor:Peptide-Major Histocompatibility Complex (TCR:p-MHC) Binding

**Figure 3:**
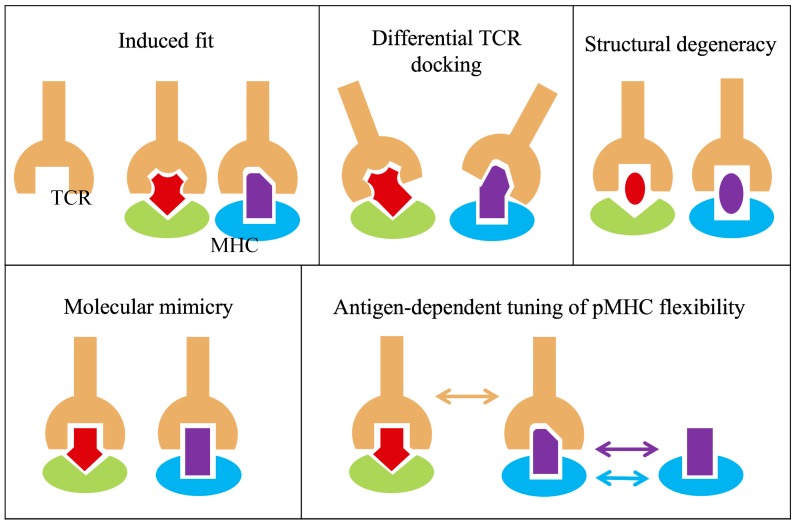
Revisiting T cell receptor-peptide major histocompatibility complex (TCR-pMHC) binding, possible molecular mechanisms of T cell recognition of cross-reactive peptide epitopes. Adapted from [42].

**Figure 4:**
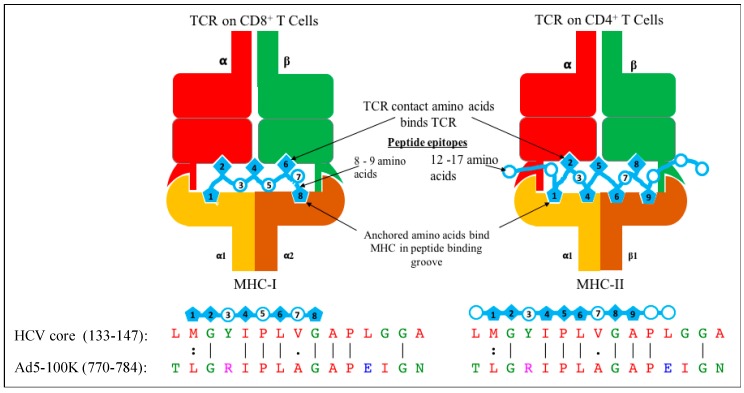
Putative model of CD8^+^ and CD4^+^ T cell receptor recognizing heterologous HCV core peptide (133–147) and Ad5 100K protein epitope (770–784) in context of MHC.

The basic principle of recognition of an antigen peptide in the context of MHC molecules by the T cell receptor was initially suggested to be based on stringent interactions. This stringency supports the clonal selection theory of T cells and the one peptide-one T cell clonotype paradigm, even though the T cell receptor (TCR) only contacts one to three amino acids of a peptide bound to MHC [41]. However, based on this strict specificity of TCR, it has been rather difficult to explain the mechanisms of positive and negative selection in the thymus where not all the exhaustible repertoire of potential peptides is present, there can be induction of autoimmunity due to environmental factors not attributable to specific molecular epitopes, allo-reactivity occurs, etc. [39,41]. Consequently, the concept of T cell cross-reactivity has arisen, and due to its significance, mechanisms to understand the phenomenon of cross-reactivity have been extensively investigated. At a molecular level, cross-reactivity of T cells can occur by multiple strategies including conformational plasticity of TCR-CDR (Complementarity-Determining Region) loops, altered TCR:p-MHC docking geometry, structural degeneracy, molecular mimicry, and/or flexibility of the peptide and MHC binding (Figure 3) [42,43]. Based on these molecular mechanisms, peptides from related or unrelated antigens with very little homology have the potential to stimulate cross-reactive T cells. Further, a given TCR may use a combination of these molecular mechanisms to exponentially increase the diversity of antigen recognition and efficiency of our adaptive immunity [43]. These multiple mechanisms, however, make it difficult to perceive an in silico technological platform that might feasibly allow the prediction of cross-reactive T cell epitopes. Thus, predicting cross-reactive T cell epitopes is, at present, largely an empirical science. A putative model is presented for recognition of heterologous HCV core peptide (133–147) and Ad5 peptide (Ad5-100K (770–784)) with ~53% homology by a single TCR of CD8+ and CD4+ T cells in context of MHC molecules (Figure 4).

## 6. Physiological Role of Heterologous Immunity

**Figure 5:**
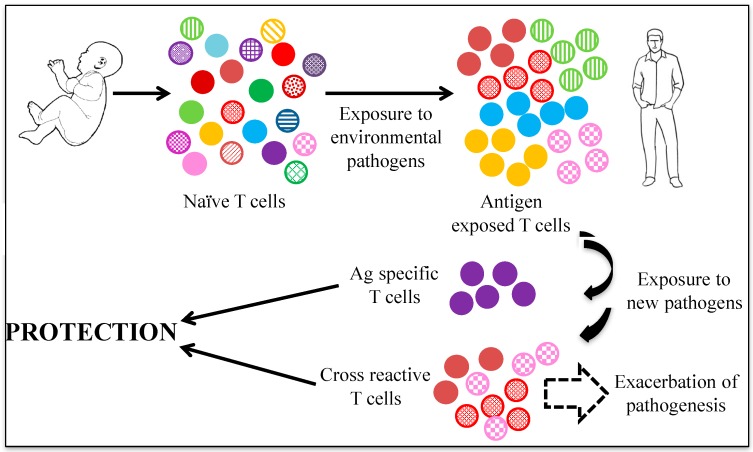
Heterologous immunity plays a significant role in protection from a new infection.

Heterologous immunity defined by cross-reactive T cells is a very important facet of immune responses. In adults with thymic involution and absence of large-scale de novo naïve T cell production, it could be extremely formidable if specific peptide epitope-specific clonotypes of T cells must be located and primed upon exposure to a new pathogen (Figure 5). In contrast, tackling a new infection with an army of cross-reactive T cells would be highly efficient and quick. Our immune system is continuously exposed to environmental microbes and pathogens to such an extent that no human adult is immunologically naive [40]. This results in the establishment of a sizeable pool of memory T cells. Immunity to ubiquitous microbes could be a major mechanism in evoking populations of memory T cells that are cross-reactive against a pathogen in a naive individual, modulating the outcome of a new infection [39,40,41]. They could modulate the breadth of the T cell repertoire, influence the memory T cell pool and/or the immune-dominance of specific epitopes, and could act as a double-edged sword resulting in enhanced or diminished immune responses against a new pathogen [39,40,41]. Physiologically, there is a vast imbalance in peptide-MHC binding and TCR specificity. Approximately 10^15^ peptides can be presented by self MHC, whereas <10^8^ T cell clonotypes with a total of ~10^12^ T cells/host exist in humans. This suggests that each TCR must recognize multiple (~10^6^) peptide epitopes [35]. Therefore, it is expected that a large number of cross-reactive T cells exists, but they have been neither extensively characterized nor exploited for human health. Beyond the physiological framework, heterologous immunity can reduce or enhance the effectiveness of vaccines by skewing the induced T cell responses. It can also form the basis of peptide-dependent interventions to prevent severe pathology caused by autoimmunity, and could also be important in designing new unconventional vaccines targeting antigens that are not strictly derived from a given pathogen. We also speculate that targeting heterologous immunity may be a significantly better approach to designing immunotherapeutic vaccines against chronic infections such as HIV, HCV, HBV, tuberculosis (TB), etc., since in most of these cases, antigen-specific T cells have been made anergic, modulated, suppressed, and/or exhausted due to continued exposure to the pathogen. In this regard, a non-recombinant non-replicative adenovirus could serve as a potential vaccine for HCV. 

## 7. Implications of Heterologous Immunity on HCV Infection: Beyond Unexpected Observations

**Figure 6:**
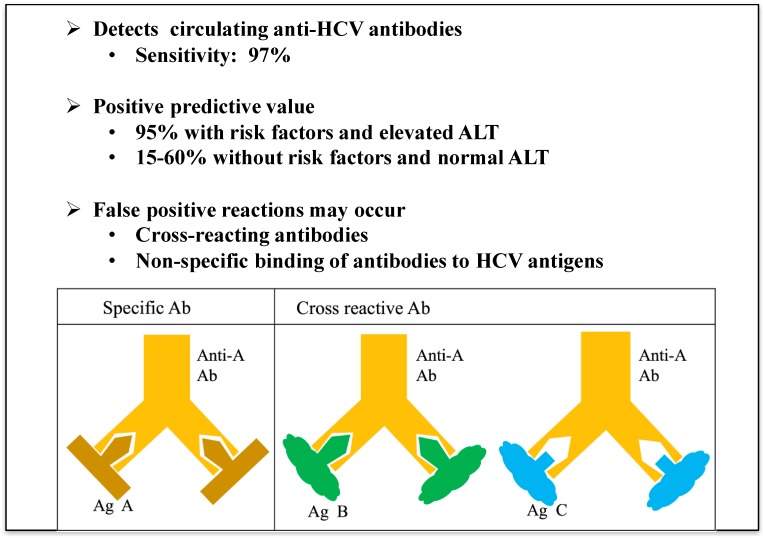
Diagnosis of hepatitis C virus by anti-HCV antibodies Enzyme Immunoassay or Chemiluminescence Immunoassay (EIA or CIA).

It can be speculated that robust heterologous immunity between adenoviruses and hepatitis C virus may affect multiple aspects of HCV diagnosis, infection, and progression of chronic disease in manners both beneficial and detrimental to the host. It has been reported that HCV diagnostic tests using anti-HCV CIA (Chemiluminescence immunoassay) or EIA (Enzyme immunoassay) lead to an average of 35% (range between 15% to 60%) false positive results among low risk group of immunocompetent individuals, but the reasons are not known (Figure 6) [44]. Although our studies focused more on cross-reactive cellular immune responses, when we examined antibody responses in both humans and mice, we found evidence of cross-reactive anti-HCV antibodies (using recombinant HCV antigens obtained from Chiron) in the presence of anti-Ad antibodies [34]. It remains to be seen whether false positive anti-HCV antibodies, observed in low-risk healthy humans by diagnostic EIA and CIA assays, correlate with seropositivity to Ad viruses (Box 1). Further, due to the ubiquitous nature of infections with adenoviruses in humans, it remains to be established whether the two natural courses of HCV infection, acute or chronic, are dictated at least in part by pre-existing T cell immunity to Ad in humans. Large prospective and/or retrospective studies with human subjects can shed light on this important aspect of heterologous immunity and perhaps resolve the longstanding enigma of acute vs. chronic HCV infection. It is not clear, however, why extensive studies of human cellular immune responses against HCV antigens, both in HCV-unexposed and HCV-exposed subjects, have not revealed the cross-reactivity between Ad and HCV that we have observed. Regardless, such cross-reactive immune responses can explain the high prevalence of false positive diagnostic tests, spontaneous re-activation of HCV-specific functional immune responses and viral clearance, and the cases of HCV-specific memory T cells in HCV-naïve individuals. It remains to be established, however, whether and how pre-existing immunity to adenoviruses shape the course of natural infection with HCV in the human population. Furthermore, since recombinant adenovirus vectors are being tested as candidate vaccines for several different pathogens including Ebola virus, Plasmodium, mycobacteria, influenza viruses, among others, their widespread use as vaccines could significantly impact the prevalence and natural course of HCV infection [45].

Box 1. **Future directions and implications of heterologous immunity on HCV.**

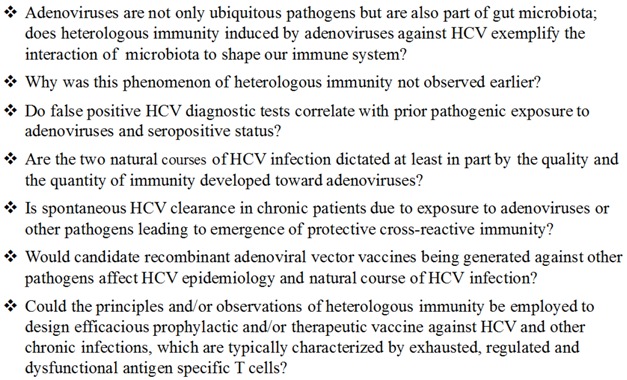

## Figures and Tables

**Table 1:** Peptides derived from various HCV proteins contain amino acid sequence homology with Ad proteins.

HCV Proteins *	No. of HCV Peptides Tested	No. of Ad Proteins Tested	Number of Peptides (15-20 Amino Acid Long) Showing Homology (% Homology between HCV Peptide and Ad Protein Sequences)					
			25.00–30.00	30.11–35.00	35.11–40.00	40.11–45.00	45.11–50.00	>50.00
Core	45	27	45	43	27	0	7	1
F	16	27	16	15	4	0	3	1
NS3	11	27	10	8	8	1	1	0
NS4	20	27	15	7	2	0	0	0
NS5a	29	27	29	10	7	2	0	0
NS5b	39	27	39	7	4	1	0	0

Sequence alignments were performed using ClustalW software. * HCV antigens E1, E2, P7, and NS2 show low homology (<25%) with the lowest number of Ad proteins and are not shown here. Adapted from [34].

**Table 2:**
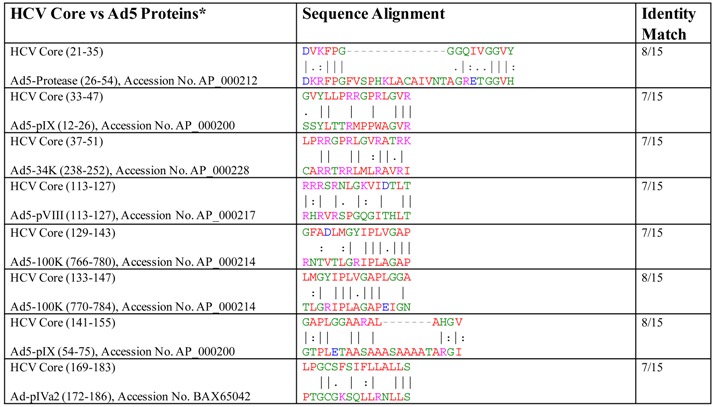
Amino acid sequence alignment of representative HCV core peptides (15 aa long) with various Ad5 proteins (Clustal-W alignment score > 45).

* Protein sequence identifier for Ad5 proteins are shown in the table. A | (vertical line) indicates positions with fully conserved residue. A : (colon) indicates conservation between groups of strongly similar properties. A . (period) indicates conservation between groups of weakly similar properties.
